# Platelets in HIV: A Guardian of Host Defence or Transient Reservoir of the Virus?

**DOI:** 10.3389/fimmu.2021.649465

**Published:** 2021-04-23

**Authors:** Etheresia Pretorius

**Affiliations:** Department of Physiological Sciences, Stellenbosch University, Stellenbosch, South Africa

**Keywords:** HIV-1, platelet, platelet complexes, receptors, thrombotic risk

## Abstract

The immune and inflammatory responses of platelets to human immunodeficiency virus 1 (HIV-1) and its envelope proteins are of great significance to both the treatment of the infection, and to the comorbidities related to systemic inflammation. Platelets can interact with the HIV-1 virus itself, or with viral membrane proteins, or with dysregulated inflammatory molecules in circulation, ensuing from HIV-1 infection. Platelets can facilitate the inhibition of HIV-1 infection *via* endogenously-produced inhibitors of HIV-1 replication, or the virus can temporarily hide from the immune system inside platelets, whereby platelets act as HIV-1 reservoirs. Platelets are therefore both guardians of the host defence system, and transient reservoirs of the virus. Such reservoirs may be of particular significance during combination antiretroviral therapy (cART) interruption, as it may drive viral persistence, and result in significant implications for treatment. Both HIV-1 envelope proteins and circulating inflammatory molecules can also initiate platelet complex formation with immune cells and erythrocytes. Complex formation cause platelet hypercoagulation and may lead to an increased thrombotic risk. Ultimately, HIV-1 infection can initiate platelet depletion and thrombocytopenia. Because of their relatively short lifespan, platelets are important signalling entities, and could be targeted more directly during HIV-1 infection and cART.

## Introduction

**Figure 1:**
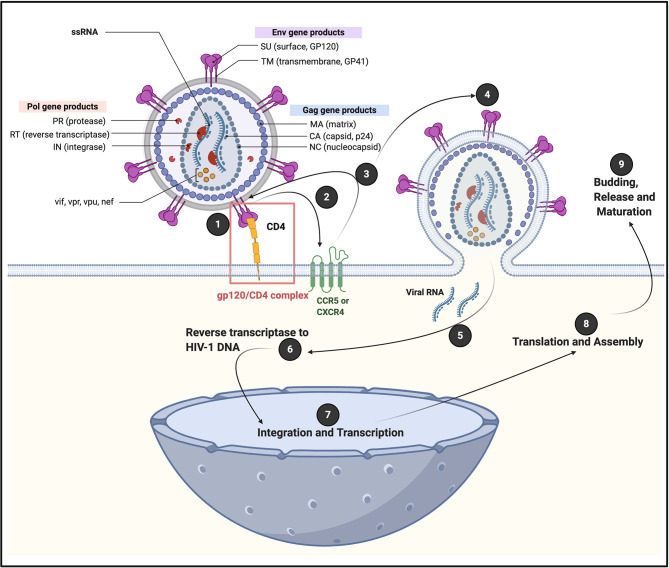
A generic illustration of the initial attachment of HIV-1 to a CD4^+^ cell, resulting in in a series of sequential steps that allows viral replication. Initial HIV-1 cell contact result in the interaction of viral envelope glycoprotein gp120 with CD4 receptors, to form a (1) gp120/CD4 complex on the host cell surface (4). This interaction induces a conformational change in the envelope protein that exposes a chemokine receptor binding site. (2 and 3) Association of gp120 with chemokine receptor CC chemokine receptor 5 (CCR5) or chemokine receptor 4 (CXCR4), promotes a rearrangement of the transmembrane envelope protein gp41, resulting in the (4) fusion of the viral and cellular membranes and the entry of the viral capsid into the cell (4–6). CXCR4 and CCR5 were initially identified for their role in HIV-1 entry of CD4^+^ T cells through its interaction with gp120 (7). CCR5 is a G protein-coupled receptor (8), with seven transmembrane segments and an eighth α-helix parallel to the plasma membrane (6). (5) Viral RNA is now released into the cell, followed by (6) reverse transcriptase to HIV-1 DNA; (7) integration and transcription in the nucleus; (8) translation and assembly in the cell cytoplasm; followed by (9) budding and release and maturation. Diagram created with BioRender (https://biorender.com/).

Globally, human immunodeficiency virus 1 (HIV-1) 38.0 Million people are living with HIV (WHO and UNAIDS 2019 data) and this number have increased with 24% relative to 2010 (1). HIV-1 expresses structural genes (*gag, pol*, and *env*), regulatory genes (*rev* and *tat*) and accessory genes (*vpu, nef*, *vpr*, and *vif*) (2). The various gene products drive virus infection in cells that express CD4 (cluster of differentiation 4) membrane glycoprotein receptors on their plasma membranes (3). After initial attachment of HIV-1 to the CD4 receptor, a series of sequential steps will follow, resulting in viral replication (see **Figure 1**). HIV targets are mainly CD4^+^ T cells, macrophages and dendritic cells; however, it can also pursue CD8^+^ T cells, B cells and natural killer (NK) cells (9), haematopoietic progenitor cells, astrocytes, platelets (10–12), macrophages and monocytes (13) and can also engage with neutrophils (14). Interestingly, megakaryocytes, but not platelets, express the CD4 receptor for HIV-1 attachment (15). However, platelets do have various other receptors that can directly bind to either intact HIV-1, or to its envelope protein inflammagens (16).

The immune and inflammatory responses of platelets to HIV-1 and its envelope protein inflammagens are of great significance to both the treatment of the infection itself, and to the comorbidities related to systemic inflammation (12). Platelets play crucial roles in primary haemostasis and thrombosis. In addition, their complex reactions to viral (and bacterial) signals result in immune responses, and may be protective, or may contribute to significant systemic inflammation (17–24). Platelet receptors allow them to survey and interact with signals from pathogens (pathogen-associated molecular patterns; PAMPs) and also signals from damaged cells (damage-associated molecular patterns; DAMPs) (24). Platelet receptor interactions with PAMPs and DAMPs result in platelet-platelet, platelet-leukocytes or platelet-erythrocyte aggregates, leading to their depletion (25) and eventually thrombocytopenia (24). Typically, platelets have a lifespan of 8 to 10 days, but in HIV-1 infection this lifespan might be halved (26) or even decreased by two thirds (27). Thrombocytopenia in HIV-1 infected patients can be the result of a combination of shortening of platelet life span, doubling of splenic platelet sequestration, as well as direct impairment in platelet formation by HIV-infected marrow megakaryocytes (27). It is also known that megakaryopoiesis may be altered during the course of HIV-1 infection (28) and affect the erythroid lineage (29). Immune thrombocytopenia purpura may also occur in HIV-1 infection (30, 31). Platelets (with viral loads) are cleared from the circulation and have shortened survival rates, which ultimately also result in thrombocytopenia (32–34). Additionally, platelets can adhere to endothelial cells, creating an adhesion molecule-dense area with which leukocytes can interact and perform immune functions (35).

Platelets can interact with the HIV-1 virus itself or with viral proteins like Tat (transactivator of transcription), or with inflammatory molecules in circulation due to HIV-1 infection. These various interactions may lead to four distinct physiological processes:

1. The inhibition of HIV-1 infection *via* endogenously-produced inhibitors of HIV-1 replication.
2. HIV-1 can temporarily hide from the immune system inside platelets, whereby platelets act as HIV-1 reservoirs. The use of combination antiretroviral therapy (cART) has significantly reduced mortality and morbidity in HIV patients (36). However, during cART interruption, HIV-1 may re-appear from HIV-1 reservoirs within platelets, resulting in viral persistence. This phenomenon may have significant implications for treatment.
3. HIV-1, its envelope protein inflammagens and also circulating inflammatory molecules from the disease and its comorbidities, can trigger platelet complex formation and hypercoagulation.
4. Sustained HIV-1 infection may result in platelet depletion and eventually thrombocytopenia.

## Platelets Interact Directly With HIV-1

**Figure 2:**
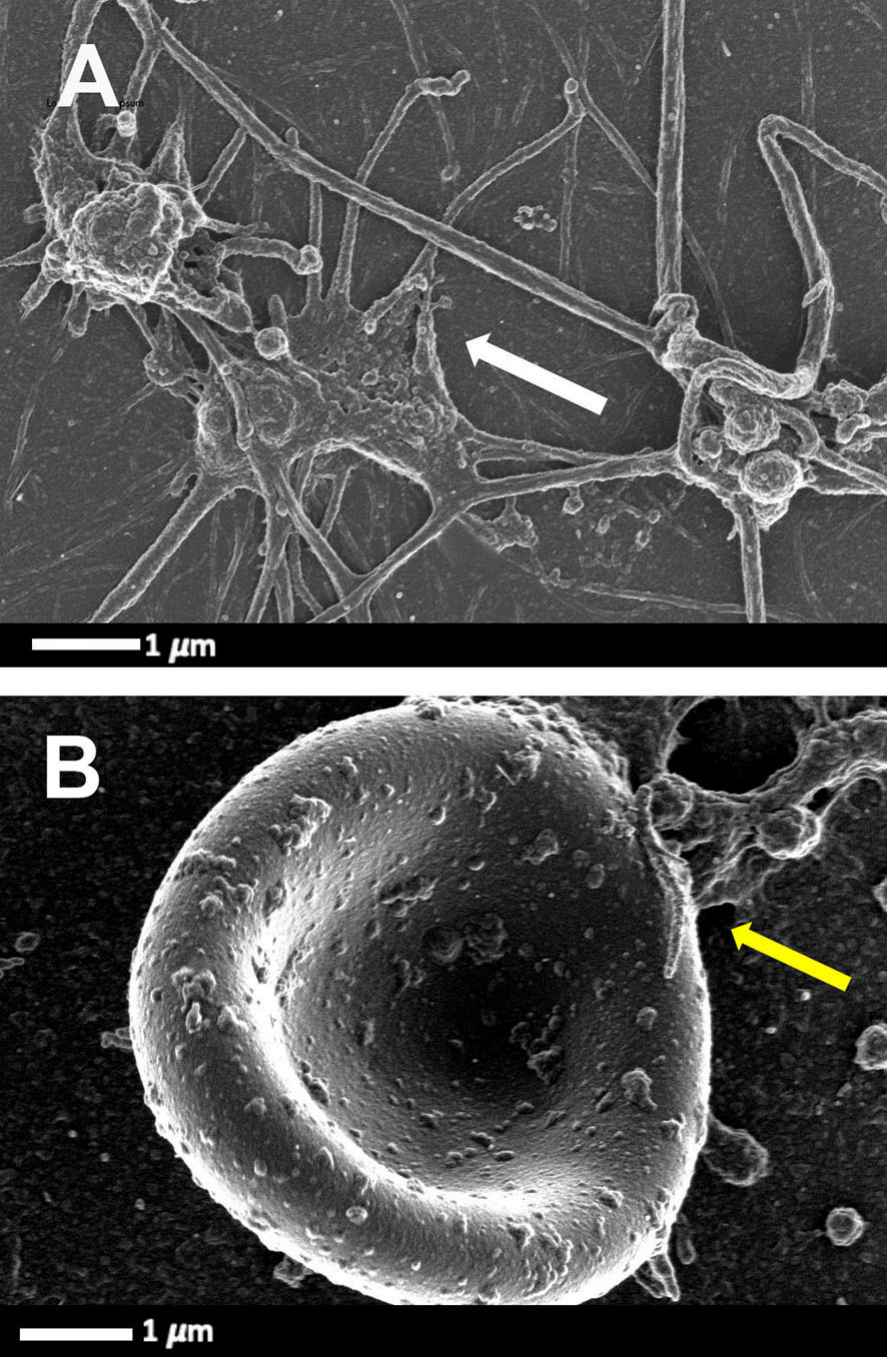
Scanning electron microscopy micrograph plate from patients with HIV and with deep vein thrombosis and on primary treatment (emtricitabine, tenofovir and efavirenz) (cART). **(A)** Hyperactivated platelets with pseudopodia, spreading and microparticle formation (white arrow). **(B)** Platelet-erythrocyte complex, yellow arrow: platelet forming pseudopodia that attaches to an erythrocyte membrane (raw data taken from (42).

Platelets greatly contribute to host defence by multiple mechanisms, including forming immune complexes and aggregates, shedding their granular content, internalising pathogens and subsequently being marked for removal. The process whereby platelets internalize HIV-1 was first described in 1990 (37). Platelets can also activate and recruit leukocytes to sites of infection and inflammation, and modulate leukocyte behaviour to support the leucocyte’s ability to phagocytose and kill the virus. Direct HIV-1 binding and interactions with platelets lead to platelet (hyper)activation (38), microparticle formation (39, 40), platelet reactivity (41) and aggregation to themselves, to blood vessels, immune cells and also to erythrocytes. **Figure 2** is a scanning electron microscopy micrograph plate showing hyperactivated platelets and an erythrocytes-platelet complex in patients with HIV-1; raw data taken from (42). Platelet-erythrocyte complexes are known to bind HIV-1 (18, 43).

### Platelet Receptors Bind HIV-1 Directly

**Figure 3:**
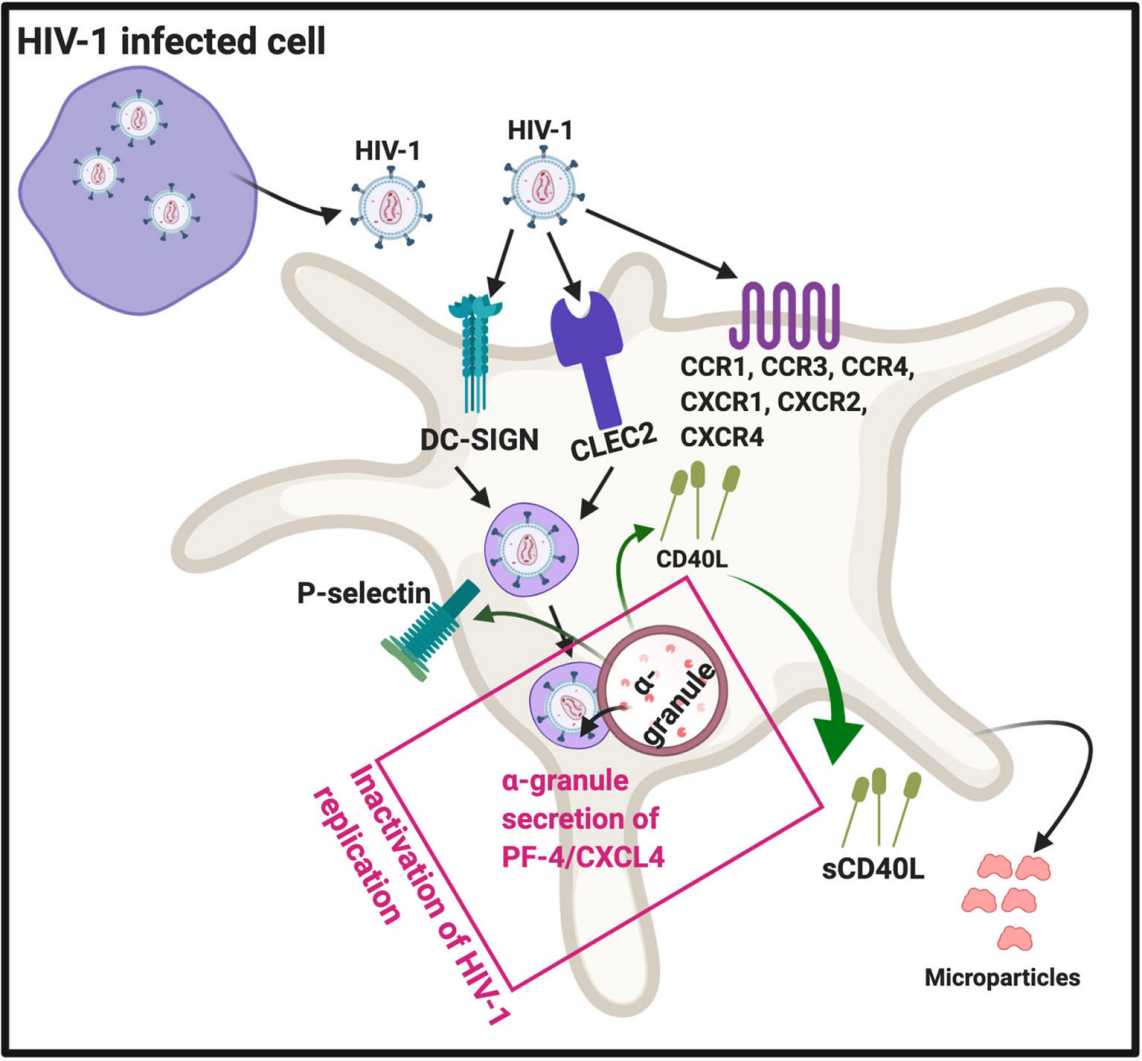
HIV-1 interact with platelets, resulting in (hyper)activation and microparticle formation. Platelet receptors that are known to bind viruses (16): C-C chemokine receptor type 1, 2 and 4 (CXCR1, CXCR2, CXCR4), as well as C-C chemokine receptor type 1, 3 and 4 (CCR1, CCR3 and CCR4). Diagram created with BioRender (https://biorender.com/).

There are four subfamilies of chemokine co-receptors, CC, CXC, CX_3_C and XC (44) and they are part of the G-protein coupled receptor family that are integral membrane proteins. Platelets express various chemokine co-receptors (45, 46). In platelets, chemokine receptor CXCR1, CXCR2, and CXCR4, as well CCR1, 3 and 4 can directly bind to HIV-1 (16). Some of these co-receptors can also be transferred to HIV-negative cells through platelet microparticles (18). It was found that microparticles derived from both platelets and megakaryocytes containing the co-receptor CXCR4, and may transfer CXCR4 to CD^+^/CXCR4-null cells (47). This process may play an important role in spreading HIV-1. DC-SIGN (a C-type lectin receptor) is also present on platelets (48) and can bind HIV-1 (16, 17). In addition, platelets express CLEC2 (a C-type lectin-like receptor), a well-known activation and modulating platelet receptor (49). CLEG2 is also a receptor for HIV-1 (16, 50, 51). See **Figure 3** for a simplified diagram that shows platelet/HIV-1 interactions.

After platelets internalize HIV-1, the virus can either be sheltered (unaltered), with potential transfer of the virus into target organs, or they can come in contact with platelet secretory products. Intact HIV-1 are present in enclosed endocytic vesicles, were they are sheltered from platelet secretory products. However, HIV-1 that are present inside the surface-connected canalicular system, have been in contact with platelet secretory products (52). Platelet secretory products, mainly from α-granules, may lead to its destruction and ultimately facilitated platelet clearance (52). Activated platelets can inhibit HIV-1 replication through the secretion of platelet factor 4 (PF-4) (also known as CXCL4) (53). PF-4 is a chemokine that is stored in platelet α-granules (17), see **Figure 3**.

### Platelet Complex Formation Due to HIV-1

Platelets have the ability to form platelet-cell complexes with various circulating blood cells, including with each other, CD4^+^ and CD8^+^ T cells, neutrophils monocytes and also macrophages. These complexes are mediated by membrane-membrane interactions *via* receptor binding. Platelets can also form complexes with adhesive proteins including fibrinogen and von Willebrand factor (VWF) (54). Such platelet-cell and platelet adhesive protein complexes form part of platelet activation mechanisms and vascular remodelling (54) and impacts on platelet structure, granule secretion, surface glycoprotein expression, and molecular activation pathways of platelets (24, 55).

During HIV-1 infection, activated platelets can also form aggregates, conjugates or complexes with CD4^+^ and CD8^+^ T cells (56, 57), and in particular with memory T cells that are HLA-DR^+^ and CD38^+^ (17). HLA-DR and CD38 are activation markers on T cells during HIV-1 infection (58). Platelets with engulfed virus particles may also form aggregates with CD16^+^ inflammatory monocytes (17). Human monocytes are classified into two subtypes, based on the expression of CD16: classical CD14^+^CD16^−^ monocytes and the proinflammatory CD14^+^CD16^+^ monocytes (59).

Funderburg and co-workers in 2012 found that in HIV-1 infection, non-classic (CD14^+^CD16^++^) and intermediate (CD14^++^CD16^+^) monocytes are increased and also express high levels of tissue factor and P-selectin (CD62P) (60). P-selectin is another protein that is central in facilitating complex formation between platelets and T cells, and platelets and monocytes. P-selectin expression on platelet membranes is a very complex process, and can also result due to platelet activation by dysregulated proinflammatory molecules in circulation. After virus endocytosis, platelets express P-selectin on their membranes. Simpson and co-workers in 2020 found that platelet activation can enhance viral uptake, as well as facilitates the transfer of infectious virus from platelets to susceptible CD4^+^ T cells (57). This happens in part because of the expression of surface-bound P-selectin, that drives platelet-CD4^+^ T cell complex formation (57). When P-selectin is present on platelet membranes, it acts as receptors that are able to bind to P-selectin glycoprotein ligand-1 (PSGL1) on T cells. PSGL1 protein is expressed by all T cells; however, the affinity to bind its ligand is determined by the degree of glycosylation. PSGL1 is not functional in naive T cells (61). Platelets are also recognised by macrophages, causing platelet clearance from the circulation. Platelet clearance may ultimately result in thrombocytopenia, which is a major complication of HIV-1 infection (62–64). Phagocytosis of platelets by macrophages depends on surface exposure of the phosphatidylserine (PS) and clustering of GPIbα, but neither one appears involved in binding (65). Possible candidates for P-selectin-independent binding to macrophages are CD36, the αvβ3 vitronectin receptor, and the ligand receptor pair CD40-CD40L (65). Zapata and co-workers in 2014, mentioned that viral activation of platelets induces an increased expression of P-selectin that functions as a receptor for macrophages; and platelet-leukocyte aggregation may result inf phagocytosis by macrophages (66). HIV-1 may also activate platelets to express P-selectin which then acts as a receptor for macrophages (52). Circulating platelets bound by autoantibody are also targeted for removal by low-affinity Fc-receptors predominantly expressed on splenic macrophages (67). FcγRIIa, as well as other Fc-receptors on macrophages mediates platelet phagocytosis and clearance from the circulation (68, 69).

Soluble (s)CD40L is increased during HIV-1 infection (70), and present in plasma of HIV patients (71). Elevated sCD40L may induce immunosuppression during HIV infection (72). CD40 is a glycoprotein and a member of the tumour necrosis factor superfamily and is found on the cell surface, either as a monomer, a dimer or trimer (73). It is expressed on the surface of activated T cells and involved in complex formation between platelets and immune cells. Despite the conventional association of CD40 expression with CD4^+^ T cells, there are reports that shows that CD8^+^ T cells are likewise capable of expressing CD40L (74).

CD40L originates from platelet α-granules. CD40L exists either as a transmembrane form or a soluble form (75). CD40L is released from platelets following activation by thrombin, ADP, or collagen (76). After its release from the α-granules, it migrates to the platelet membrane. It can also be shed as sCD40L which can then in turn, bind to the receptors CD40, αIIbβ3, α5β1, or Mac-1 (neutrophil integrin α_M_β_2_) (77). CD40–CD40L coupling plays a crucial role in different aspects of the immunity system, such as the activation of kinases (73). Both the receptors αIIbβ3 (76), and CD40 are also expressed on platelet membranes (77), and sCD40L in circulation can in turn also activate platelets (78). When sCD40L binds to the platelet α_IIb_β_3_ receptor, it promotes platelet spreading and thrombus formation, as the process allows for the migration of P-selectin to the platelet membrane. The P-selectin on the membranes of sCD40L-activated platelets can form complexes with monocytes *via* the P-selectin receptor on the platelets and PSGL-1 on the monocytes (78).

**Figure 4:**
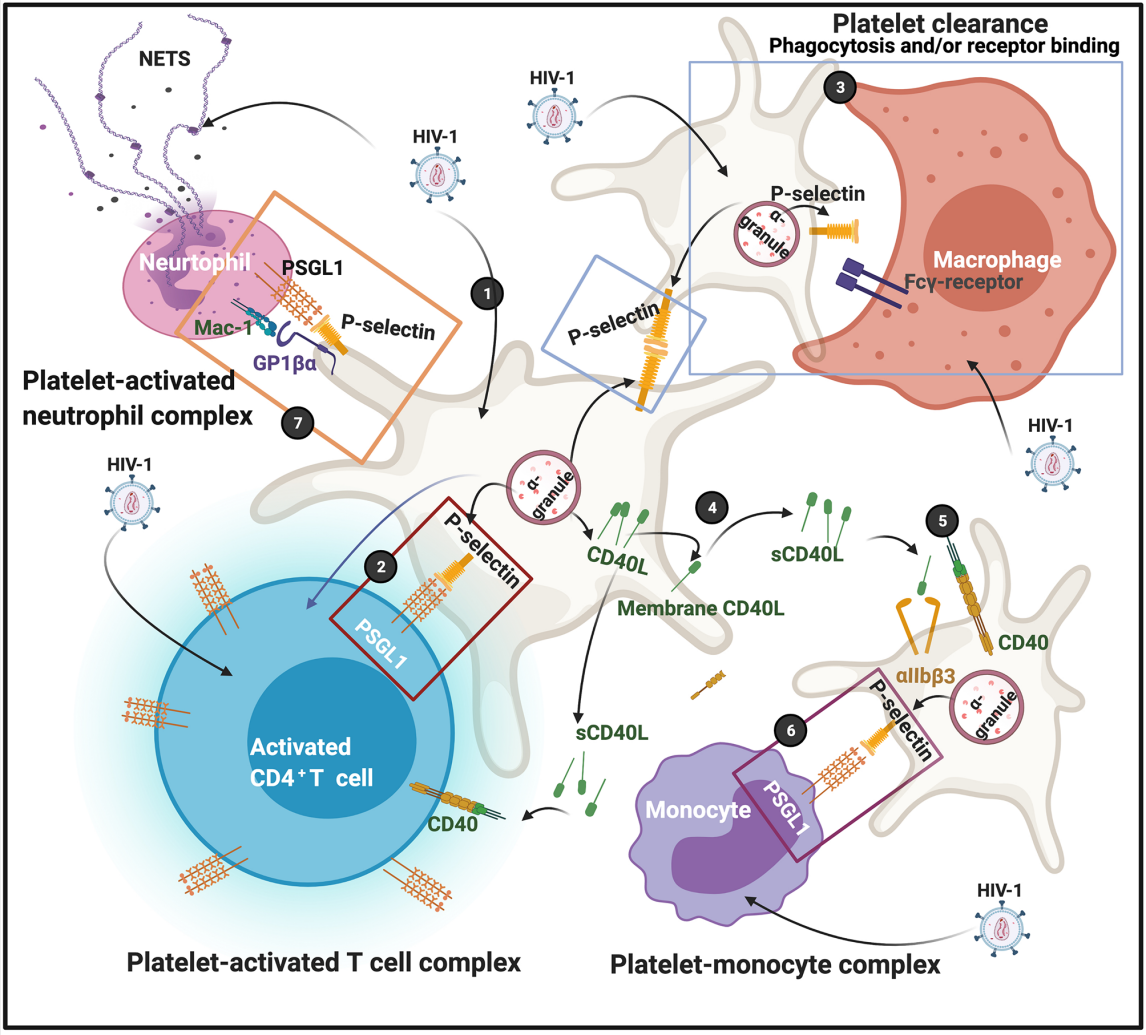
(1) After virus endocytosis, platelets express P-selectin on their membranes, followed by platelet-T cell complex formation (2); P-selectin on platelet membranes are also recognized by macrophages, possibly by the Fcγ-receptor; clearance may result due to either receptor binding or phagocytosis (3). CD40L is released from platelets and can migrate to membranes or shed as soluble (s)CD40L (4). sCD40L can bind to both the α_IIb_β_3_ or CD40 receptors (5) The P-selectin on the membranes of sCD40L-activated platelets can also form complexes with monocytes (6). Platelet-neutrophils also form complexes (7) Diagram created with BioRender (https://biorender.com/).

Platelet-neutrophil complexes is the result of platelet glycoprotein Ibα (GP1bα) binding to Mac-1 or because of platelet P-selectin binding to neutrophil PSGL-1 (79). In addition, integrin α_IIb_β_3_ also serves as a binding partner for Mac-1 on neutrophils *via* a bridge of soluble fibrinogen (80, 81). Neutrophils detect HIV-1 by Toll-like receptors (TLRs) TLR7 and TLR8, which recognize viral nucleic acids (82). Downstream effects of the platelet-neutrophil interaction result in amongst others, generation of neutrophil extracellular traps (NETs). NETs trap pathogens (including HIV-1), preventing their amplification and dissemination (83). Recently it was also found that NETs may restrain HIV-1 production in macrophages (84). See **Figure 4** for a simplified diagram that shows platelet complex formation.

## Hiv-1 Proteins and Their Binding to Endothelial Cells and Platelets

Platelet hyperactivation and endothelial cell damage are closely linked. The interface of platelet-endothelial cell interactions result in the release of various inflammatory and mitogenic substances. These substances alter the chemotactic, adhesive and proteolytic properties of endothelial cells (85). Tat (trans-activator of transcription) play major roles in both platelet activation (86) and endothelial dysfunction (87, 88). When Tat and gp120 binding happens, inflammatory and mitogenic substances are released. Tat enhances viral transcription (89), and has been detected in the sera of patients with HIV (90) even during cART (91). It is released from cells with active HIV-1 replication, or from latently HIV-1-infected cells into neighbouring uninfected cells, even in the absence of active HIV-1 replication and viral production due to effective cART (92). Activation by Tat requires the chemokine receptor CCR3 and β3-integrin expression on platelets, as well as the activation of a calcium flux. In turn, Tat binding to platelet receptors causes platelet microparticle formation (16) and sCD40L release (93) [sCD40L in turn drives cellular complex formation (as described earlier)].

### Endothelial Cells and HIV-1 Protein Interactions

Endothelial damage and dysfunction is a risk factor for cardiovascular events in HIV-1 (94). Although HIV-1 itself do not actively replicate in endothelial cells, endothelial dysfunction depends on the release of both HIV-encoded proteins, as well as inflammatory mediators into the microenvironment by HIV-infected cells (87). Because endothelial function, structure and healthiness are closely linked to platelet functions, and because Tat can trigger endothelial dysfunction, this section briefly discussed endothelial cell and HIV-1 protein interactions.

Tat and the envelope glycoprotein, gp120 are actively secreted into the endothelial cell micro-environment during HIV infection (87). Tat can bind to the integrin receptor α_v_β_3_ on endothelial cells to trigger endothelial dysfunction (95). Urbinati and co-workers in 2012 found that immobilized Tat induces actin cytoskeleton organization, formation of α_v_β_3_ integrin(+)focal adhesion plaques, and recruitment of vascular endothelial growth factor receptor-2 (VEGFR2) in the ventral plasma membrane of adherent endothelial cells (96). Tat binding to the endothelial cells may also directly contribute to atherosclerosis and cardiovascular disease in patients with HIV (97).

**Figure 5:**
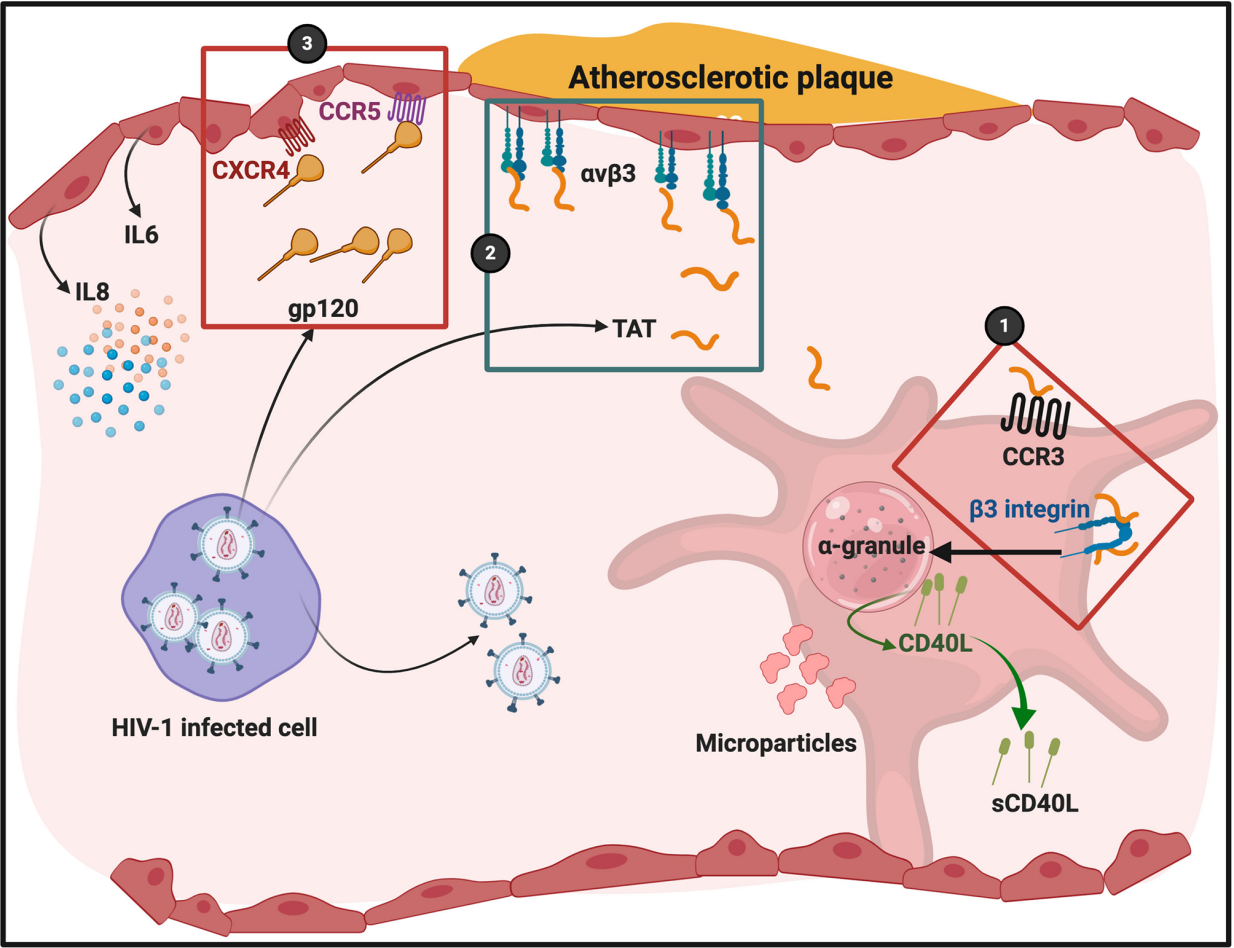
HIV-1 and its trans activating factor (Tat) particles interacting with platelets and endothelial cells. Atherosclerotic plaque formation is known to cause endothelial damage and shown here to indicate an area of endothelial damage. Tat is expressed by HIV-1 infected cells and activates platelets through chemokine receptor CCR3 and integrin β3 (86) (1). Tat binds to endothelial cell integrin receptor α_v_β_3_ (2). gp120 binds to endothelial cell receptors CXCR4 and CCR5 (3). Diagram created with BioRender (https://biorender.com/). Cells are not drawn to scale.

gp120 can also bind to CXCR4 and CCR5 on endothelial cells. When gp120 bind to these receptors, it potentially might promote endothelial cell senescence. Hijmans and co-workers in 2018 showed that HIV-1 gp120 can induce cell senescence, but the authors did not prove it was due to a direct interaction of HIV-1 with CCR5 or CXCR4 (98). Gp120 binding to endothelial cells facilitates upregulation of pro-inflammatory cytokines such as IL-6 and IL-8 (87, 99). In addition, Gp120 binding to endothelial cells also increases endothelial permeability (100) and down-regulation of tight junction proteins (101). For a detailed review of HIV-1 protein interactions with endothelial cells with the resulting pathophysiology, see (102). **Figure 5** shows a simplified diagram of HIV-1 proteins binding to platelets and endothelial cells.

## The Indirect Pathophysiology of Platelet During HIV-1 Infection

Thrombo-embolic events cardiovascular disease, and microvascular disease are well-known to occur during HIV-1 infection (42, 103–107). Cytokines and biomarkers of impaired coagulation (fibrinogen, fibrin, thrombin D-dimer and VWF) are all dysregulated in HIV-1 (108). These molecules and their pathophysiological effects on platelets during HIV-1 infection may have significant effects on platelet activation and may also drive thrombo-embolic events and microvascular disease during HIV-1 infection (109). These inflammatory molecules may be involved in both primary and secondary haemostatic events during HIV-1 infection. Primary haemostasis happens as a response to vascular injury or damage, resulting in platelets adhering to the damaged endothelial wall. Secondary haemostasis results in formation of the clot and enzymatic activation of coagulation proteins. The next paragraphs will briefly discuss events involving circulating inflammatory biomarkers and coagulation proteins during HIV-1 infection.

During HIV-1 infection an exaggerated systemic inflammatory response (110) guides platelet dysfunction, where platelets are inappropriately activated, followed by immunological destruction, followed by HIV-1-related thrombocytopenia. Dysregulated inflammatory cytokines during HIV-1 infection, include IL-1 (α/β), IL-2, IL-6, TNF-α, IFN-α/β, NF-κB and IFN-γ (111). TNF-α in particular, is a prominent pro-inflammatory cytokine that plays a critical role in HIV-1 pathogenesis (112).

During HIV-1 infection, platelets attach to the exposed or damaged sub-endothelium with the platelet GPIb-IX-V receptor complex; and also with platelet GPVI adhesion receptors to exposed collagen from damaged endothelial cells (24, 35, 113). The HIV-1 matrix protein p17 (p17), secreted from HIV-1-infected cells (114) and platelets, can directly interact with the endothelium, and may cause the release of soluble endothelial pro-inflammatory molecules, including sVCAM-1, sICAM-1 and VWF. When VWF enters the circulation or attaches to areas of (damaged) vessel walls, it self-assembles into strings and fibres, enabling platelet adhesion (115). sVCAM-1, sICAM-1 and VWF are known to be elevated in circulation in HIV-1 infected patients, and their presence are associated with thrombosis (116–118). Increased circulating VWF levels have been linked to recurrent venous thrombo-embolic events in patients with HIV-1 (106). This spiral of events ultimately cause thrombocytopenia and support the development of microvascular and arterial thromboses.

Molecules like VWF, thrombin, fibrin, fibrinogen (and D-dimer, are associated with (hyper)coagulation, and closely linked to the development of coagulopathies, thrombocytopenia and microvascular disease noted in HIV-1 infections (42, 103–105). Increased D-dimer concentrations found in HIV-1 infection (119) are also associated with poor cardiovascular and other clinical outcomes in people with HIV-1 infection (120). Similarly, an increase in thrombin and coagulation factors are also present in HIV patients, while decreased levels of anti-thrombin and protein C, and increased levels of Factor V, Factor VIII, were also previously noted (121). Thrombin was also found to facilitate HIV-induced cell fusion, probably by activating gp120 (122). For a comprehensive review on the effects of hypercoagulation in HIV-1 see (24). Thrombin is a well-known activator of platelets, and can cause platelets to show Ca^2+^ influx, integrin α_IIb_β_3_ activation and phosphatidylserine exposure disintegrate into cellular fragments containing organelles, such as mitochondria, glycogen granules, and vacuoles (123). Metabolic ATP depletion and impairment of platelet contractility along with significant cytoskeletal rearrangements, also occurs simultaneously with platelet disintegration  (123). In addition, ADP plays a significant role in platelet activation. ADP-induced platelet aggregation is mediated by P2Y1 and P2Y12 G-protein-coupled receptors (124). ADP also plays a key role in platelet recruitment to the blood vessel wall, while adenosine and high concentrations of ATP inhibit ADP-induced platelet aggregation (125). These molecules also facilitate the progression of platelet activation in HIV-1 infection (125). Platelet aggregation play a key role in cardiovascular events. It has been shown that the integrase inhibitor raltegravir (RAL) may reduce persistent HIV-induced platelet hyperreactivity and aggregation (126).

## Platelets During HIV-1 Treatment

A variety of cART drug therapies are currently available by prescription and their main goal is to prevent the virus from replicating and reduce viral load, thereby reducing to possibility of transmission of HIV-1 to others. These therapies also aims to restore CD4 counts and immune function, to reduce comorbidities from HIV-1, and to ultimately improve survival rate. Research shows that platelets from patients with HIV-1 still show hyperactivation, even while they are on cART drug therapies. Platelets derived from HIV-infected individuals under stable cART exhibit a phenotype of increased activation, activation of the intrinsic pathway of apoptosis and undermined granule secretion in response to thrombin (127).

HIV-1 reservoirs are significant obstacles in HIV-1 treatment and eradication. These reservoirs allow persistence of replication-competent HIV-1 for prolonged periods of time in patients on optimal cART regimens (128). The main HIV cellular reservoir is composed of resting CD4^+^ T-cells (129), and unfortunately, replication-competent provirus from latent reservoirs is capable of reigniting infection, if therapy is interrupted (130). Peripheral Vγ9Vδ2 T cells are a novel reservoir of latent HIV-1 infection (131). It is also known that megakaryocytes can also contain HIV-1 and that these cells may play a role in persistence of HIV-1. HIV-1 was also shown to integrate in terminally differentiated astrocytes, suggesting that astrocytes could be a permanent reservoir of provirus in brain (132, 133). HIV-1 may also be present in cerebrospinal fluid (CSF) (134), and can replicate there, with development of CSF HIV-1 resistance resulting in acute or subacute neurological manifestations (135).

Macrophages also harbour HIV-1 DNA, but whether this reflects active infection or phagocytosis of infected CD4^+^ T cells is still not clear (128). It has now also been reported that human platelets harbouring HIV-1, can indeed spread the virus to macrophages. Real and co-workers in 2020 isolated platelets from patients with HIV-1, and on antiretroviral therapy (12). Fifteen of the patients had a detectable viral load in their blood, whereas in 27 individuals, the viral load was below the detection limit (12). The authors found that 10 of the 27 patients with suppressed viral load had detectable HIV RNA in their platelets, and further analysis showed that these platelets indeed contained intact virions. Megakaryocytes also contained HIV-1, suggesting that these cells were the likely origin of the platelet-associated virus (12). In addition, incubation of platelets from these patients with a reporter cell line, showed that the virus was indeed replication competent. This study confirmed that platelets may be transient carriers of HIV-1 and may provide an alternative pathway for HIV-1 dissemination in HIV-infected individuals on cART with viral suppression, and poor CD4^+^ T cell recovery (12). These results are particularly significant as it shows that platelets with replication-competent HIV-1 can propagate infection to macrophages (10–12). However, is should be noted that the viral reservoir in platelets may be limited (about 10 viral RNA copies per million of platelets) (11). The HIV-1 reservoir in platelets is therefore small in comparison with latently infected cells in lymphoid tissues. In a 2016 viewpoint paper, various experts in the field discussed the constitution of HIV-1 viral reservoirs, how to measure the various reservoirs’ viral content, and how best to eradicate reservoirs (128). In this 2016 review paper, it was stressed that the only true HIV-1 reservoirs, are resting CD4^+^ T cells (128).

## Conclusion

Platelets are now recognized to play a complex and dynamic role in HIV-1 infections, as they act as both the guardians of host defence, as well as transient reservoirs of the virus. During HIV-1 infection viral envelope protein inflammagens and numerous inflammatory cytokines shed in the inflammatory HIV-1 milieu, have a severe impact on platelet function, ultimately leading to platelet hyperactivation, clearance and eventually thrombocytopenia. Their role in platelet complex formation can also contribute to pathophysiological inflammatory processes, endothelial dysfunction, arthrosclerosis and immunopathology. Although lower platelet counts are associated with worse prognosis, platelets may also be a transient reservoir for HIV-1.

Because of their relatively short lifespan, platelets are important signalling entities and could be targeted more directly during HIV-1 infection and cART, to closely evaluate and track the course of the infection. Novel approached like transcriptomics and single-cell monitoring could enable new discoveries on how platelets (and megakaryocytes) function in human health and disease (136). Although relatively low viral copies have been found in platelets (on average 9.92 HIV RNA copies per million platelets) (12); therapies targeting specifically platelets during HIV-1 infection could possibly prevent HIV-1 hiding in them. In addition therapies that might prevent platelet hyperactivation and ultimately thrombocytopenia, could also have an impact on the effects of platelet depletion, noted during HIV-1 infection.

## Author Contributions

The author confirms being the sole contributor of this work and has approved it for publication.

## Conflict of Interest

The author declares that the research was conducted in the absence of any commercial or financial relationships that could be construed as a potential conflict of interest.
